# *In Vitro/In Silico* Study on the Role of Doubling Time Heterogeneity among Primary Glioblastoma Cell Lines

**DOI:** 10.1155/2017/8569328

**Published:** 2017-10-31

**Authors:** M.-E. Oraiopoulou, E. Tzamali, G. Tzedakis, A. Vakis, J. Papamatheakis, V. Sakkalis

**Affiliations:** ^1^Department of Medicine, University of Crete, Heraklion, Greece; ^2^Computational Bio-Medicine Laboratory, Institute of Computer Science, Foundation for Research and Technology-Hellas, Heraklion, Greece; ^3^Neurosurgery Clinic, University General Hospital of Heraklion, Heraklion, Greece; ^4^Gene Expression Laboratory, Institute of Molecular Biology and Biotechnology, Foundation for Research and Technology-Hellas, Heraklion, Greece; ^5^Department of Biology, University of Crete, Heraklion, Greece

## Abstract

The application of accurate cancer predictive algorithms validated with experimental data is a field concerning both basic researchers and clinicians, especially regarding a highly aggressive form of cancer, such as Glioblastoma. In an aim to enhance prediction accuracy in realistic patient-specific environments, accounting for both inter- and intratumoral heterogeneity, we use patient-derived Glioblastoma cells from different patients. We focus on cell proliferation using* in vitro* experiments to estimate cell doubling times and sizes for established primary Glioblastoma cell lines. A preclinically driven mathematical model parametrization is accomplished by taking into account the experimental measurements. As a control cell line we use the well-studied U87MG cells. Both* in vitro* and* in silico* results presented support that the variance between tumor staging can be attributed to the differential proliferative capacity of the different Glioblastoma cells. More specifically, the* intratumoral heterogeneity* together with the overall proliferation reflected in both the* proliferation rate* and the* mechanical cell contact inhibition* can predict the* in vitro* evolution of different Glioblastoma cell lines growing under the same conditions. Undoubtedly, additional imaging techniques capable of providing spatial information of tumor cell physiology and microenvironment will enhance our understanding regarding Glioblastoma nature and verify and further improve our predictability.

## 1. Introduction

Glioblastoma (GB), a grade IV glioma as categorized by the World Health Organization (WHO) [1], is one of the most aggressive brain cancer types [2] with a poor prognosis for the patient [3], despite the rapid advances in technology and novel therapeutics. One of the most characteristic features of GB that limits therapeutic potential is heterogeneity [4]; both different molecular GB subtypes [5, 6] and subclonal cell populations coexist within the same tumor [7–9]. Hence, the importance of individualized GB treatment and understanding of patient-specific GB pathophysiology is evident and research plans towards this aim are of great interest.

The use of the widely scientifically studied common GB cell lines passaged in lab conditions for decades [10] is nowadays questionable with respect to their clinical relevance in therapeutic outcome prediction and to their ability of representing the extensive heterogeneity observed among patients [11]. To this front, a common GB trend is the use of patient-derived GB cells to enable preclinical physiologic estimations and personalize therapeutic strategy. Basic researchers cooperate with clinicians in order to isolate GB cells and promote the establishment of short-term primary GB cell cultures [12–15], which provide additional results back to the patient. Established methods for biological research and early drug discovery utilize cell lines grown on plastic culture flasks. Over the years, the ability of these* in vitro* systems to provide biologically relevant answers and describe drug effects is limited due to the fact that they are too simplistic and do not include key players of the phenomenon. Hence, researchers seem to mobilize more realistic experimental approaches such as 3-dimensional (3D) cell cultures [16–20] and/or* ex/in vivo* implantations [14, 21–23] to better imitate cancer in a mechanistic and conditional way. Biological 3D models comprise an important step to describe the early phases of tumor progression before going to the complexity of* in vivo* systems.

Biological experiments are strongly linked with computational and mathematical (*in silico*) models.* In silico* models offer a systematic framework of understanding the underlying biological processes integrating knowledge and information from multiple biological experiments and/or clinical examinations [24]. By predicting the behavior of the system, new targeted experiments can be designed. In that way, the process of mathematical modeling validation is an iterative refinement procedure [25], which terminates when a valid and biologically plausible and concrete description of the system that reproduces the observed cellular behaviors and growth patterns is found. Several mathematical approaches have been proposed to describe the complex, multiscale spatiotemporal tumor evolution. According to their mathematical perspective, these approaches can be classified into continuum and discrete models. Continuous mathematical models are commonly used to describe tumors at tissue level focusing more on the collective, averaged behavior of tumor cells [26–28]. On the other hand, individual-cell-based models using discrete and hybrid discrete-continuous (HDC) mathematics can describe the behavior of each cancer cell individually as it interacts with its microenvironment. Individual-cell-based models are in general more suitable to describe* in vitro* experiments, animal models, and small-sized tumors [29–34].

In general, such mathematical models attempt to translate tumor physiology hallmarks [35] into computational parameters and the predicted output is subsequently validated using as ground truth either the experimental [36, 37] or the clinical results [38, 39]. As it is well-understood, both cell division and local spreading are responsible for cancer expansion [40, 41] comprising the most important aspects for cancer progress [30, 42].* Doubling time* is defined as the average duration of cell growth and division as reflected by the cell cycle “clock” [43]. GB tumors have a remarkable rapid growth that has a critical role regarding the space-occupation and the development of intracranial pressure, usually the main reason of the GB symptomatology [44]. In previous studies, the significance of the proliferative rate has been shown. More specifically, in [45], the proliferation rates of different breast cancer patients are estimated from subsequent Magnetic Resonance (MR) images in conjunction with a simple logistic tumor growth model and show that the proliferation rate estimates could discriminate patient's survival and response to therapy. In another study [46], the role of experimental and simulated diffusion gradients in 3D tumors affecting nutrient, oxygen, and drug availability within the tumor and subsequently controlling cell proliferative rate is examined. A mathematical model parameterized from monolayer experiments is used to quantify the diffusion barrier in 3D experiments. In the recent study [40], acquisition of physiologic parameters from multicellular tumor spheroids including proliferation and death spatial profiles is used to constrain and parametrize a mathematical agent-based model that addresses several cell growth mechanisms necessary to explain the experimental observations and reductively translates them to tumor progress over time.

This work utilizes primary tumor cells collected from GB patients and subsequently cultivated* in vitro* as 3D tumor spheroids. As an initial step towards understanding the GB heterogeneity among patients, we focus on proliferation. The aim of this work is first to mathematically study the important components affecting the growth dynamics of tumor spheroids when motility is inhibited, mainly including the inter- and intratumoral heterogeneity with respect to cell proliferation and, second, to parametrize the mathematical model based on experimentally estimated parameter values of primary GB cell lines in order to increase clinical relevance. Doubling times and the average cell sizes of in-house-established primary GB cell lines from three different patients are used. The well-known U87MG GB cell line is also used as control in the experiments. All the biological experiments are performed simultaneously under the same initial and growth conditions. A hybrid, individual, cell-based mathematical model is used to predict the growth curves of the tumor spheroids and parametrized based on the experimental data. Variations in several mathematical model parameters are explored in order to quantify their effect on tumor growth expansion. The simulated results are compared to the experimental data from the relevant 3D cell cultures and show that, in combination with the proliferation rate, additional factors like the mechanical cell contact inhibition are necessary to predict the* in vitro* evolution of the different GB cell lines under study.

## 2. Methods

### 2.1. Sampling Procedure

Brain tissue sample is collected from the lesions during biopsy or gross resection of patients with indications of GB based on symptoms and MR images, while still naïve from treatment and later histologically proved to be GB cases. For the purposes of this study, we used the primary cells of three different patients. The first is a 70-year-old male patient with de novo GB close to the left brain motor area, also called GBP03 cells. The second, called GBP06 cell line, was collected from a 47-years-old female patient with a tumor in the medulla proven to be a secondary GB, which was gradually evolved to grade IV from lower grades within a time period of approximately 20 years. The third sample, called GBP08, was provided by a 53-year-old male patient with also primary GB in the temporal-occipital left hemisphere. All samples are anonymously provided with the informed patients' consent by the Neurosurgical Clinic of the General University Hospital of Heraklion, Crete, Greece, while the protocol has been approved by the Institutional Ethical Committees. Because of the relatively low success rate of the primary cell culture establishment, we are limited to these three GB cases for this work.

### 2.2. Primary Cell Cultures

Later to tissue sampling in saline solution, the specimens are immediately transferred to the lab where they are mechanically dissociated into smaller parts and supplemented culture medium is added (Dulbecco's modified Eagle medium (DMEM) with 10% fetal bovine serum (FBS) and 1% gentamycin). After gradually removing all cell debris and dead tissue parts, cancer GB cells are cultured as monolayers in standard lab conditions.

As explained before, there is much heterogeneity between GB cases and the protocol of tissue handling is slightly modified per case. An ectopic, subcutaneous implantation to immunodeficient mice is a procedural step that takes place whether the conditional stability cannot be preserved* in vitro* so that it cannot be assured that the isolated GB cells will survive and proliferate in flask. Therefore, lab animals serve as “living incubators” and usually, after the first implantation, the cells are collected and recultured until the cell culture is successfully established. In this work, GBP03 cells are passaged once, while GBP06 and GBP08 cells are directly used. All possible steps are taken to avoid animal suffering at each stage of the experiments.

### 2.3. Doubling Time Assay

We use the GBP03, GBP06, and GBP08 primary cell lines as well as the U87MG cells (ATCC® HTB-14™, USA) as control line. In order to measure the doubling time intervals of the different cell types used we apply a simple protocol in adherent cultures. In a 24-well plate, 20000 cells/ml of supplemented DMEM are seeded per cell type at day zero. The plate is incubated in standard lab conditions for approximately a week. Whenever needed, cell culture medium is carefully renewed avoiding the adherent (active) cell population to be disturbed.

Every 24 hours after seeding, the culture medium of one well per cell type is removed and trypsin-EDTA (Sigma-Aldrich, Germany) 1x solution is added for 1-2 minutes. After another 1 minute of trituration in order to produce a single cell solution, all the context is removed from the well and is transferred to a 2 ml Eppendorf tube. As a final step, 4% formaldehyde is added to permanently fix the cells within the tube which is stored to the refrigerator for further use. The procedure is repeated up to the point that 100% cell confluence is achieved. The cell concentration for each cell type is measured with a 24-hour interval by using a hemocytometer.

### 2.4. Cell Size Estimation

A divided Petri dish is plated with a single cell solution of ~2000 cells/ml and is incubated in standard lab conditions overnight to let the cells adhere in the surface of the dish. Accordingly, brightfield images of attached single cells are captured in 40x magnification and known acquisition parameters to an inverted light microscope (Leica, Germany). To check size and shape homogeneity between each cell population so that to assure that the estimated average cell size will be representative, we capture a photograph of a single cell solution within the fixed grid dimensions of the hemocytometer.

### 2.5. D Spheroid Generation

We use the hanging-drop technique in order to produce spheroids from each cell type, as recommended in [16, 17, 47]. A single cell solution of 625 cells/50 ul of supplemented double-filtered DMEM is initially seeded per well in a 96-well hanging-drop plate (3D Biomatrix, USA). Two rows of wells per cell type are plated so that approximately 24 spheroids are produced. Agarose solution of 1% w/v is added to plate's reservoirs to prevent evaporation of the droplets. After 2–4 days of cells aggregating at the bottom of each droplet, we can consider that the spheroids are finally formed. The growth progress of the spheroids is monitored over time via photographs taken under set acquisition parameters to an inverted light microscope (Leica, Germany) for predecided critical time points (2-day interval).

### 2.6. Data Analysis

The average doubling time of each cell line is estimated using exponential linear regression on the doubling time data. The average cell size of each cell line is estimated by segmenting the area of approximately 10 randomly selected cells in brightfield images to ImageJ [48] and averaging. The tumor expansion of the 3D spheroids is again estimated based on the area shown in their brightfield images. The growth curve is estimated by the mean area value ± standard deviation over time. All the above measurements are evaluated per cell type and many experiments are performed for each cell type.

### 2.7. Computational Model Implementation of Tumor Spheroids

A simplistic HDC mathematical model is used to describe the observed tumor growth of the 3D* in vitro* experiments. In the context of the HDC model, each individual cell is described by a discrete cellular automaton, while the local microenvironment is approximated by partial differential equations (PDE). In the following, a concise description of the HDC model is provided, while more thorough description can be found in [49].

#### 2.7.1. Computational Domain

To simulate a central slice of the 3D* in vitro* tumor spheroids, we set up a 2D regular lattice of size *L* = 5 mm. We assume that each *h* × *h* square lattice site fits a single cell; thus the lattice site defines the cell size as well. The same lattice is used by both the discrete and the continuous compartments.

#### 2.7.2. Continuous Compartment

For simplicity, we assume that oxygen is the only limiting molecule required by the cells in order to proliferate. The spatiotemporal evolution of oxygen is described by the partial differential equation (PDE) shown in (1). Oxygen is assumed to diffuse through the domain with diffusion coefficient *D*_*o*_, decays naturally at a rate *a*_*o*_, and is consumed by the tumor cells at a rate *γ*_*o*_. The term *c*(*i*, *j*) is 1 if there is a tumor cell at the location *i*, *j* or 0 otherwise.

(1) $$\begin{array}{c} \frac{\partial o\left(x,y,t\right)}{\partial t}=D_{o}\nabla^{2}o\left(\;x,y,t\;\right)-c_{i,j}\gamma_{o}-\alpha_{o}o\left(\;x,y,t\;\right). \end{array}$$

#### 2.7.3. Discrete Compartment

Each tumor cell is an individual entity with its own traits. Sets of these traits are assumed to represent a cellular phenotype. A more detailed description of the cell life cycle can be found in [49, 50].

**Figure 1:**
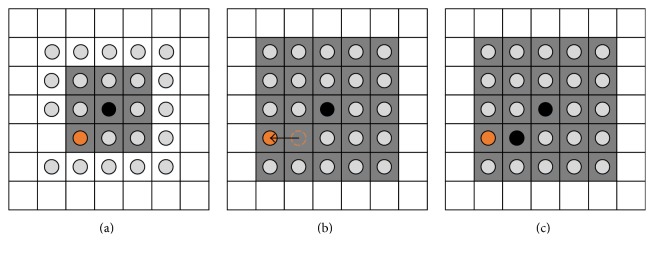
Example of a cell (shown in black) attempting to proliferate. Firstly, the cell searches the 1-Moore neighborhood highlighted by the gray squares in (a). Being unable to find an empty space, it searches the 2-Moore neighborhood indicated by the gray squares in (b) and (c). As an empty space is found, the orange cell is pushed towards the empty space as shown in (b). The latter movement frees the empty space on the 1-Moore neighborhood and allows the proliferating cell to place an identical cell (also shown in black) to the adjacent empty space (c).

In this work, two mechanisms of tumor cells are mainly considered: proliferation and death. Cellular movement has been neglected considering that the protocol of the* in vitro* experiments does not conditionally allow cell motility. Cells die if the local oxygen concentration drops below a defined threshold *o*_deadly_. When a cell dies, its location is immediately treated as empty space. On the other hand, the live cells incrementally prepare for proliferation at every time step, until the cell age reaches their doubling time. At that moment, the cell searches for a nearby empty space at the 1-Moore neighborhood. If no empty space is available, the search is expanded to the 2-Moore neighborhood (see Figure 1) and the process is repeated up to *r*-Moore neighborhood, where *r* is defined as the proliferation depth and determines the maximum neighborhood size. Examples of Moore neighborhood can be seen in Figure 1. If more than one empty space is found in the same neighborhood, one of them is randomly chosen.

As shown in Figure 1, when an empty space is found on a neighborhood other than the 1-Moore, cells are pushed away from the location of the proliferating cell towards the empty space in order to create an empty space to the 1-Moore neighborhood. Then the cell resets its cell age and places a copy of itself at the adjacent empty space. If no empty space has been found, the cell enters a quiescent state at which it constantly searches for empty space, without further increasing its age. The extended proliferating rim describes the maximum distance over which a cell is capable of pushing other cells away in order to create space for its proliferation and reflects the mechanical growth inhibition processes observed in growing cell populations [40].

## 3. Results 

In this work, the* in vitro* estimated doubling times and cell sizes of three in-house-established primary GB cell lines, as long as of the U87MG cells, are used to initialize the individual-cell-based mathematical model in an attempt to predict their different growth patterns. A sensitivity study is performed where the effects of important factors affecting tumor spheroid expansion such as the doubling time, the cell size, and the depth of the proliferative rim and the coexistence of multiple clones with different proliferative capacities within the tumor are computationally explored. We argue that, as expected, proliferation is one of the most defining characteristics regarding tumor expansion and that tumor predictive computational models should prioritize these remarkable variances between individuals and not just based on theoretically defined values.

### 3.1. *In Vitro* 2D Cultures

#### 3.1.1. Cell Size Estimation

**Figure 2:**
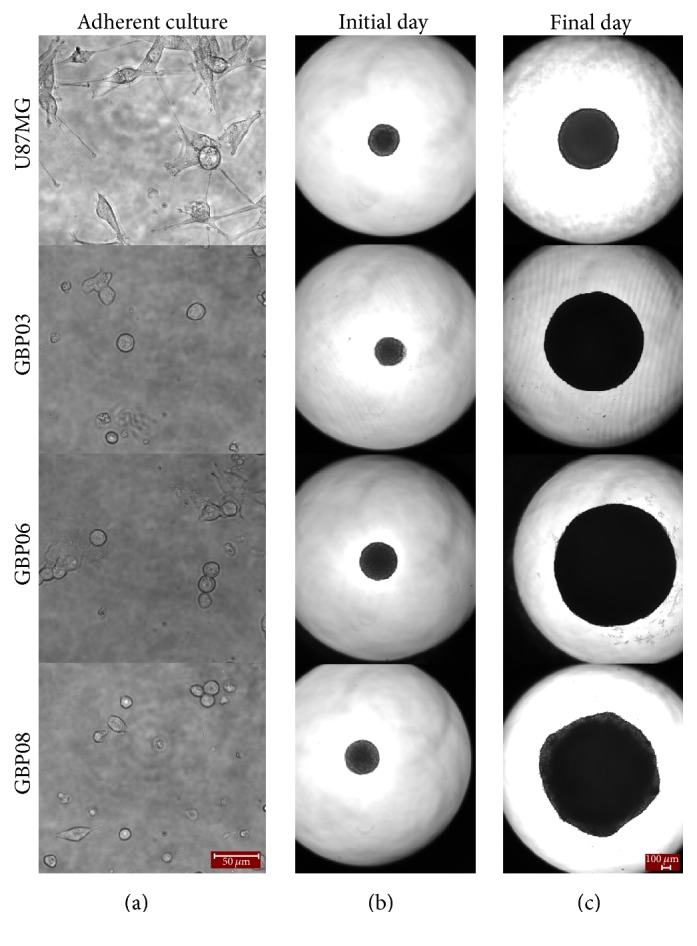
U87MG cells along with primary GB cells growing as monolayers ((a) 40x magnification) and as hanging-drop spheroids (initial day in (b) and final day in (c), 4x magnification). Scale bars are 50 and 100 microns, respectively. The initial day is set to be the first day of cell aggregation in spheroidal shape after seeding, meaning Days 2–4. Accordingly, the final day is the time point where spheroids start to deform and decompose, usually approaching well's borders. This day is Day 14 for most primary spheroids.

A usual answer of what a common human (cancer) cell diameter could be is about 10 to 100 microns [51, 52], and actually, most computational approaches assume cell size within 10–30 microns [29]. In 2D cultures of low confluence, the cell size and shape are in resting state and not crucially influenced by neighboring cells. As depicted in Figure 2, there is much homogeneity in U87MG culture with the cells conforming a rather prolonged typically observed shape, with a soma cell size varying between 19 and 24 microns in diameter (see also Table 1). On the contrast, all primary cells used in our study are smaller and typically round with not many cellular protrusions compared to U87MG cells, yet cells of the same cell line appear to differ within the same population. In case of U87MG cells, it is expected that after all these years in lab conditions there is not much morphological diversity within the cell population and that the cell soma size adequately represents the cell line. On the other hand, regarding primary cells, the cell size is only an average of all possible phenotypes within each cell line. More specifically as denoted in Table 1, GBP03 cells have an average cell diameter of 19 microns, while GBP06 are approximately 16 microns and GBP08 are close to 15 microns in diameter. Also, U87MG cells, when growing in adherent cultures, intrinsically form aggregates when much confluent. On the contrary, the primary cells studied here seem to continue as monolayers no matter the level of confluence. Obviously, the average cell size of a certain cell population, no matter how well represented in 2D, it is not maintained when growing in 3D culturing since other physiological parameters that will be discussed next also affect the cell surface-to-volume ratio altering both size and shape.

#### 3.1.2. Doubling Time Estimation

Based on literature, glioma cells usual doubling time ranges from 24 h to a couple of days [53], but more often established primary GB cell lines are recorded to vary few days [12, 54, 55]. Particularly for U87MG cells, they are supposed to have a population doubling time approximating 34 hours, according to their product sheet (ATCC HTB-14, USA). Our measurements presented in Table 1 are in line with the bibliographic records. Specifically, U87MG cells have a mean doubling time of 30.8 ± 2.5 h, which is the slowest division between the cell types we use. Among the primary cell lines, GBP03 cells divide approximately every 25.4 ± 0.5 h, while GBP06 and GBP08 have similar doubling times estimated at 23.5 ± 0.7 h and 23.0 ± 1.5 h, respectively.

### 3.2. *In Vitro* GB Spheroids

The hanging-drop technique used here to generate the 3D spheroids is a method conditionally approaching the real avascular tumoral state* in vivo* [17]. The spheroid size is determined with optical microscopy and monitored over time. It should be noted that, the imaging approach used here cannot give any quantitative estimate of the compactness of the cells or any other spatial information including the number of the cells, the cell size, shape, and polarity, which are definitely different between 2D and 3D structures.

In general, we observe that both primary and U87MG cells need approximately 4 days from single cell solutions to aggregate into spheroidal structures, while during this starting period, they seem to suppress proliferation capacity. However, most often, primary cells aggregate sooner than U87MG ones after seeding.

**Figure 3:**
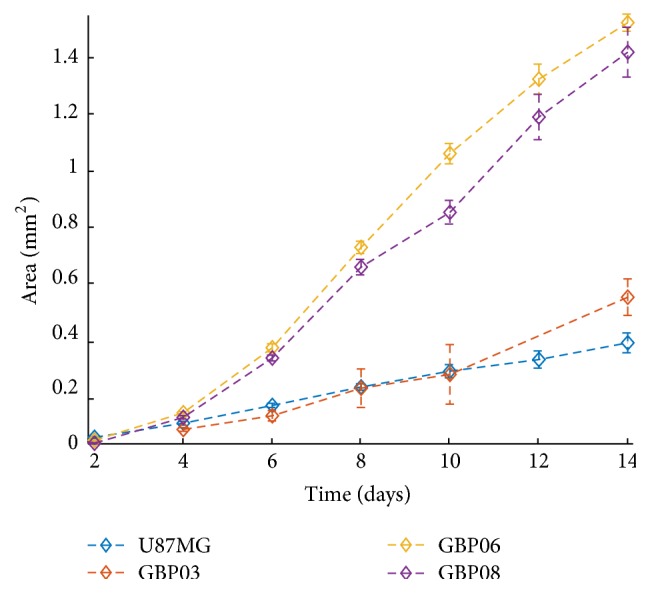
Growth of the tumor spheroid area over time for the* in vitro* experiments of each cell line.

Figure 2 illustrates the growth area of the* in vitro* spheroidal domains as imaged in 2D brightfield images at the initial and final day. The growth curves of each cell line are shown in Figure 3. An apparent difference between patients, but also between primary and conventional cell lines, can be observed. To be more specific, all primary spheroids grow larger than the U87MG cells. GBP06 and GBP08 primary spheroids follow an initial fast growing, exponential phase that slows down after approximately 6 days. U87MG spheroids have an almost linear growth pattern. It has to be clarified that the spheroids reach the well's borders before the plateau and decay phases are observed. The patients GBP06 and GBP08 adopt a high growth pattern, while the patient GBP03 follows an intermediate growth rate closer to the U87MG cell line. As already mentioned, especially for the primary cell lines, the initial distribution of the subclones, when plating the cells (Day zero), is random. This eventually leads to a multifactorial subclonal spheroid growth integrated to average estimations.

### 3.3. Computational Parameter Study

Prior to parametrizing and predicting the growth pattern of the multicellular spheroids, a simple parameter study is performed to determine the extent at which the doubling time and cell size affect the 3D growth simulation, as well as explore the effect of additional parameters that could play a significant role in tumor expansion including the depth of the proliferative rim and intratumoral heterogeneity.

The discrete and the continuous part of the computational model are parametrized accordingly to meet the experimental setup as shown in Table 2. The length *L* of the computational domain equals 5 mm to resemble approximately the size of the hanging-drop plate. Both the oxygen decay rate and the cell's oxygen consumption rate were adopted from [29]. To numerically solve the PDE (1), its parameters have been nondimensionalized by using *o*_max_, *τ*, and *L*, which correspond to the maximum oxygen concentration, the computational iteration time, and the domain length, respectively. Dirichlet boundary conditions are used to lock the boundaries to the maximum oxygen concentration to simulate the so-assumed adequate and stable nutrients' availability, since the culture medium during the experiment is periodically refreshed. Also, the alternating directions implicit method is used to numerically solve the PDE [56, 57].

**Figure 4:**
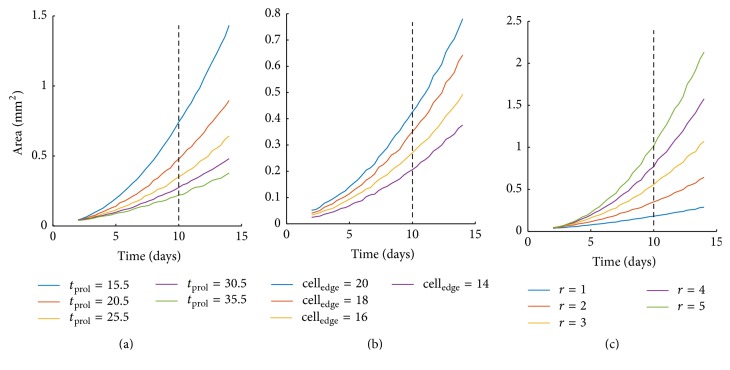
Growth of the tumor spheroid area over time as predicted from the computational model related to altering doubling time from 15.5 h to 35.5 h (a), the cell size from 14 to 20 microns (b), and the proliferation depth from 1 to 5 (c).

At first, we explore the effect of doubling time on tumor expansion keeping the rest modeling parameters constant. Specifically, we assume a tumor cell of size equal to 18 *μ*m and consider a depth of proliferative rim equal to 2 cells, while varying the doubling time from 15.5 h to 35.5 h.  Figure 4(a) shows the growth curves of the tumors with different doubling times. As expected, increased proliferative capacity results in increased tumor expansion. If a reference time point is picked at 10 days, we can calculate the absolute increase of area yielded by the decrease of the doubling time. When the doubling time is reduced from 35.5 h to 30.5 h, the area increases by approximately 24.46%; while comparing the respective areas between the doubling times 20.5 h and 15.5 h, the area is increased by 54.87%. We can thus conclude that the expansion area is affected more, when the doubling times are lower. As expected, the effect is accumulative; thus if a later/earlier time point was picked the differences would increase/decrease, respectively.

We also explore the effect of cell size on the observable tumor expansion. It should be noted that if counting of the tumor cell population was possible on the* in vitro* experiments, then this parameter would make no difference. We vary the cell size from 14 to 20 *μ*m, while keeping the doubling time constant and equal to 25.5 h and the proliferation depth equal to 2 cells.  Figure 4(b) shows that, by increasing the cell size, the tumor expansion increases as well, as expected. Indicatively, by comparing the values at simulation time 10 days, the area relatively increases by 21.5%, 29.8%, and 31.1% as the cell size increases from 14 *μ*m, 16 *μ*m, and 18 *μ*m to 16 *μ*m, 18 *μ*m, and 20 *μ*m, respectively.

The depth of the proliferative rim significantly affects the tumor expansion as it increases the number of proliferative cells.  Figure 4(c) illustrates the effect that different proliferation depths have on the tumor area over time. The proliferation time was set to 25.5 h and the cell size to 18 *μ*m. At the reference point of 10 days, as the proliferation depth increases from 1 to 5 cells with a step of 1 cell, the area increases relatively to its previous value by 94.7%, 58.4%, 38.9%, and 31.3%. In other words, a considerable higher expansion of the tumor area (94.7%) is observed when the proliferation depth is increased from 1 to 2, as compared to a change from depth 4 to 5. As the proliferation depth increases, less cells enter the quiescent state and proliferate instead; this is why the growth area is increased.

**Figure 5:**
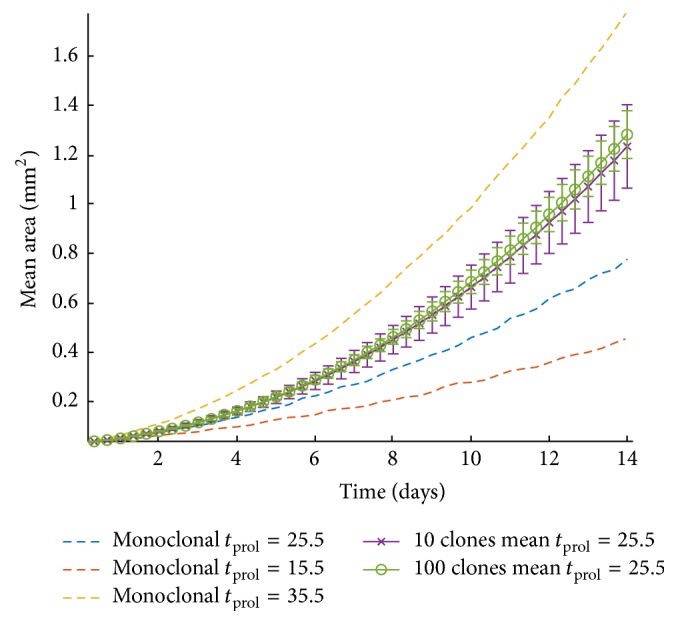
Monoclonal and polyclonal tumor area expansion. For the polyclonal case two scenarios are considered: one at which the number of phenotypes is 100 (green line) and another where 10 phenotypes are randomly selected (purple line). Each experiment is repeated 50 times and the corresponding standard deviation is also shown. The mean area of three monoclonal examples with doubling times 15.5 h (red dashed line), 25.5 h (blue dashed line), and 35.5 h (yellow dashed line) is also illustrated.

**Figure 6:**
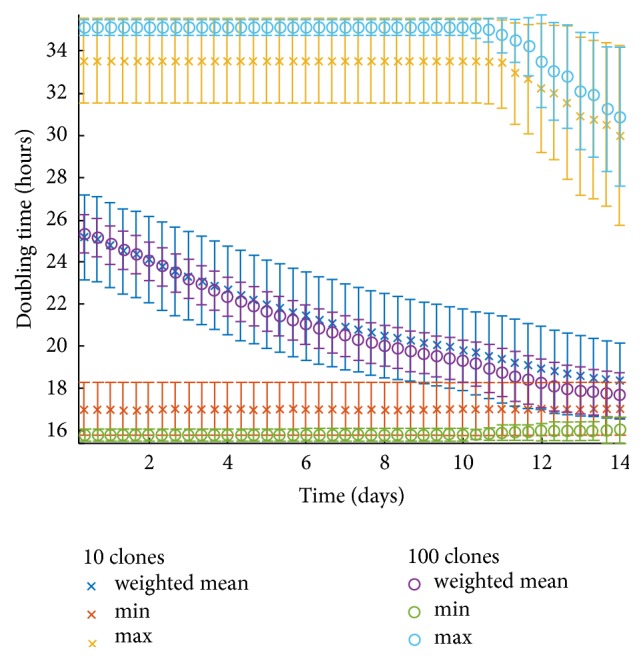
Doubling time of the populations that survive over time in a polyclonal tumor. Two scenarios are considered: one at which the number of phenotypes is 100 and another where 10 phenotypes are randomly selected. Each experiment is repeated 50 times. The minimum, maximum, and average doubling times for both scenarios are shown, as well as their corresponding standard deviations.

To further investigate the role of heterogeneity between our cases, we proceed by performing simulations which contain multiple phenotypes identical in all traits except for their respective doubling time. All phenotypes have their cell size set to 18 *μ*m and proliferation depth (*r*) equal to 2 cells. The proliferation time is randomly selected for each phenotype at the beginning of the simulation from a uniform distribution in the interval (15.5, 35.5) hours. As shown in Figure 5, to illustrate the impact of the phenotypic multitude, two scenarios are considered inspired by [29]: one at which the number of phenotypes is 100 (shown in green line) and another where 10 phenotypes are randomly selected (shown in purple line). Additionally, given the randomness of the phenotypic initialization, each experimental scenario is repeated 50 times. Figure 5 also shows the area expansion over time for three monoclonal examples with doubling times 15.5 h (red dashed line), 25.5 h (blue dashed line), and 35.5 h (yellow dashed line).  Figure 6 illustrates the doubling time of the populations that survive over time. As it can be seen, the mean minimum and the mean maximum values of the doubling time are constant for a long period of time indicating the presence of both the fastest and the slowest populations within the tumor, yet the frequency of these populations becomes progressively unequal with the fastest population to actually overpopulate within the tumor. Thus, a decline to minimum values of the mean doubling time is observed.

### 3.4. Comparison of Biological and Computational Results

In the following, we assume monoclonal populations and parametrize the mathematical model based on the estimated experimental values for the doubling time and cell size for the different GB cell lines. We also parameterize the model without taking into account the* in vitro* estimates of cell sizes and keep the cell size and all the other parameters constant in all the experiments. Parameters within the range of the experimental biological observations are chosen to achieve the best-fitting growth curves. It has to be noted that both the simulated and the biological experiments have an initial seeding population of approximately 625 cells per spheroid per cell type. The simulations show that the* in vitro* estimates of cell sizes do not improve the model predictability and that accounting only for differences in doubling time among GB lines results in very similar growth curves.

**Figure 7:**
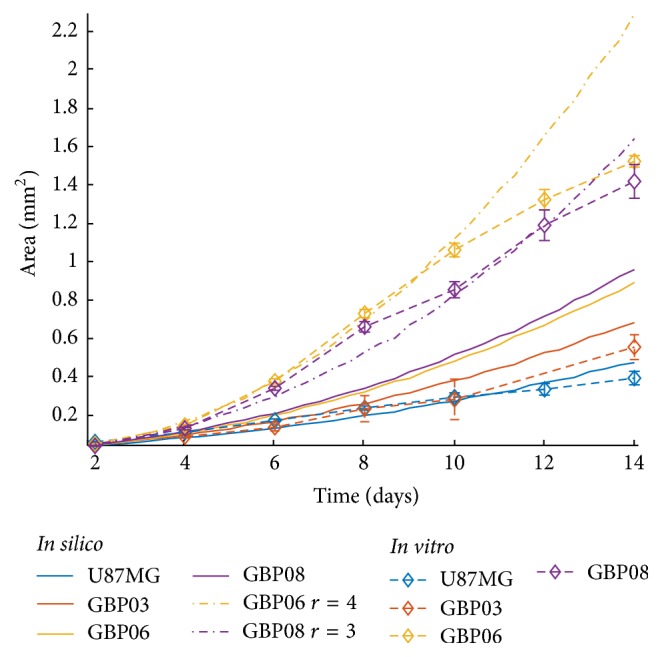
*In vitro* spheroidal growth as opposed to* in silico* for all four cell types with the final chosen sets of doubling times as shown in Table 1 and fixed proliferation depth equal to 2. Two additional simulated growth curves are depicted with different proliferation depth values for the GBP06 (*r* = 4, yellow dash dotted line) and the GBP08 (*r* = 3, purple dash dotted line) spheroids.

Table 1 shows the parameters used by the* in silico* model regarding the doubling time.  Figure 7 shows the* in vitro* growth curves and the* in silico* predicted ones for all the GB cell lines. Based on the selected doubling time values and keeping the proliferation depth equal to 2, the growth curves of U87MG and GBP03 cell lines are closely approximated by the* in silico* model. However, the GBP06 and GBP08 cell lines diverge significantly from the* in vitro *results indicating that proliferation alone is necessary, but not sufficient to explain the tumor expansion of different GB cell lines growing under the same initial conditions. Hence, additional phenomena should be taken into account. For example, increasing the proliferative depth and/or consider the possibility that multiple phenotypes with various proliferative capacities coexist within such tumors, then the* in vitro* and* in silico* growth curves would come in line as our parameter study analysis previously revealed. Alternatively one could advocate that GBP06 and GBP08 contain phenotypes with higher proliferation depth than U87MG (and GBP03) which are expected to thrive in compact environments such as a solid spheroid. It should be noted that the proliferative depth could also be affected by the development of extracellular matrix (ECM) substrate in 3D cultures, even in the conditional absence of a relevant substrate [17], as in our biological experiments. This, along with antagonistic and synergetic relationships of subclones within the growing spheroid, could alter the mechanical responses of dividing cells, reflected in terms of proliferation depth to our mathematical model. However, our biological approach did not take into account a priori this parameter, but it was the computational approach that indicates such possible behavior suggesting that ECM production and distribution might also be different in different cell lines.

Figure 7 also shows the simulated growth curves for the GBP06 and GBP08 after changing their proliferation depth values from 2 to 4 and 3, respectively. The* in vitro* data better correlate the relevant* in silico* data. Also notice that setting the proliferation depth of GBP06 higher than the GBP08 is important to achieve their corresponding growth patterns, where GBP06 grows faster than GBP08, given that the doubling time of the former is higher than the latter and that small differences in their cell sizes are not adequate to reverse their growth patterns. Another point that should be marked is that the subsequent decline observed after Day 8 in the* in vitro* growth curves of these two cell types cannot be predicted by the computational model. This is because the computational model we use does not account for inhibitory stimuli that are probably developed in real growing tumors, since this was beyond the scope of this study.

## 4. Discussion 

This work utilizes primary tumor cells collected from GB patients and subsequently cultivated* in vitro* as 3D tumor spheroids and computational approaches to study, experimentally parametrize, and predict the growth dynamics of tumor spheroids focusing on proliferation. At first, a parameter study is performed in order to evaluate the extent to which important factors such as the doubling time, the cell size, and the depth of the proliferative rim, as well as the coexistence of multiple clones with different proliferative capacities within the tumor, affect tumor spheroid expansion when motility is inhibited. The experimentally estimated doubling times and cell sizes of three in-house-established primary GB cell lines, as long as of the U87MG cells, are then used to parametrize the computational individual-cell-based model.

Overall the parameter study verifies the significant effect of proliferation (depicted in both the cellular doubling time and the depth of the proliferative rim) on tumor expansion [40] and underlines additional factors that could play an important role on tumor growth curves including the intratumoral heterogeneity that has been widely observed in GB. We also observe that a multiclonal population with the same mean proliferation exhibits a greater tumor expansion than the corresponding monoclonal population because fitter clones survive over time driving tumor expansion at higher rates. Furthermore, the clonal heterogeneity within the tumor mass allows different clones to be selected every time an experiment is performed. Thus, a variation is observed in the growth curves. The variance is cumulative, increases over time, and can reach a difference of 100 *μ*m in radius after 14 days of growth (Figure 5). Furthermore, the simulations also show that although the mean growth curves are quite similar, the variance highly depends on the initial number of different clones coexisting within the tumor mass such that fewer initial clones in the population produce higher variability (Figure 6).

Comparing the* in vitro* experiments with the* in silico* predictions, we observe that although the proliferation rate is necessary, yet it is not sufficient, to describe the growth curves we observe experimentally. The simulations show that additional factors including the intratumoral heterogeneity together with the overall proliferative capacity reflected in both the proliferation rate and the mechanical cell contact inhibition can predict the evolution of different GB cell lines. Nevertheless, further investigation of the underlying mechanisms is critical.

In general, compactness of the spheroids can be assigned to two factors in mesoscopic terms: (a) the cellularity, in means of cells' size and shape given the space, and (b) the levels of stress tolerance, reflecting their response against internal forces within the spheroid which vary between division and entering quiescence state, also known as “*contact inhibition*.” As smaller in size and quicker regarding divisions, GBP06 and GBP08 cells appear to grow larger in 3D over time than the other two cell types mainly because of their promoted proliferative capacity reflected by the higher proliferation depth in the respective simulated growth curves (see Figure 7). However, this is only an assumption for our* in silico* trials since there is no indication of spheroids cell density and proliferation depth to our experimental protocol and this is a limitation of our method needed to be taken into account in future work.

The migratory capability of our cells is conditionally blocked to our experiments so that it can be assumed to play a minor role in the proliferative characteristics studied here. However, when the different cell populations grow in 3D, both ECM can be produced, and the cell shape and polarity could also be affected, such that cell-to-cell and cell-to-matrix adhesion properties could be further explain the divergence observed over time in growth patterns between the* in vitro* and* in silico* experiments.

We suggest that, instead of using bibliographic values usually referenced by common GB cell lines, cell doubling time was found to critically enhance the* in silico* predictability but is insufficient to holistically describe differences in tumor growth over time among the different GB cell lines. The mechanical cell responses to internal forces obtained during the growth of a compact tumor should be further investigated experimentally, as well as the important role of intratumoral heterogeneity. The importance of quantitative methods to provide spatial information of proliferative, quiescent, and necrotic cells as well as additional features including the remodeling of ECM and phenotypic distribution regarding intratumoral heterogeneity affecting tumor expansion becomes evident.

## 5. Conclusions

The massive proliferation is a defining characteristic of the tumor nature, essential for its progress. When focusing on such a greed form of cancer, such as GB, constantly growing intra-axially and aggressively disturbing brain functionality, proliferation underlying processes become incompatible in cancer progress. In GB, heterogeneity is another typical hallmark, not only among patients with differences between GB molecular subtypes, but more unexpectedly, between different regions of the same tumor with the presence of intratumoral subclonal dormancies. We claim that future research should be based on primary cells directly collected from patients and that common cell lines should only serve as landmarks to unite studies of different groups. For every primary established cell line, not only molecular but also physiological parameters should be estimated to enable a more precise future clustering of different GB cases. Estimations starting with the typical doubling time as shown here and evolving to more delicate features such as delineation of necrotic and hypoxic regions or invasive capability or others are highly important. To this front, computational models may serve as predictor tools not only for estimating cancer progress [59], but also for designing targeted biological experiments. Simulations of cancer progress, either* in vitro* or* in silico*, should not anymore be based on theoretical values, especially if clinical translation is of interest. If we target the holistic description of tumor evolution, we should follow a stepwise approach, where computational tools can definitely help in identifying the most important parameters affecting the final outcome.

## Figures and Tables

**Table 1:** Mean cell sizes and doubling times (±standard deviation) as estimated from the *in vitro* experiments for the respective cell lines (first column). The *in silico* values used to initialize the HDC model regarding the doubling time are also shown.

Cell type	*In vitro* estimations		*In silico* values
	Cell diameter (*μ*m)	Doubling time (h)	Doubling time (h)
U87MG	21.5	30.8 ± 2.5	33
GBP03	19	25.4 ± 0.5	25
GBP06	16	23.5 ± 0.7	23
GBP08	15	23.0 ± 1.5	22

**Table 2:** The computational parameters used to initialize the HDC model.

Parameter	Value
Domain length, *L*	5 mm (methods-computational domain)
Cell (& lattice) size, *h*	14–20 *μ*m (methods-computational domain)
Iteration time, *τ*	8 h (methods-computational domain [49])
Oxygen consumption, *γ*_0_	6.25 10^−17 ^M cell^−1^ s^−1^ (methods-computational domain [29])
Maximum oxygen, *o*_max_	6.7 10^−6^ M O_2 _cm^−3^ (methods-continuous compartment [29])
Oxygen decay rate, *α*_0_	0.0125 (ND) (methods-continuous compartment [29, 30, 58])
